# Real-life assessment of aripiprazole monthly (Abilify Maintena) in schizophrenia: a Canadian naturalistic non-interventional prospective cohort study

**DOI:** 10.1186/s12888-019-2103-x

**Published:** 2019-04-16

**Authors:** Sally Mustafa, Joanna Bougie, Maia Miguelez, Guerline Clerzius, Emmanouil Rampakakis, Jean Proulx, Ashok Malla

**Affiliations:** 1 Douglas Mental Health University Institute, Montreal, Quebec Canada; 2 Lundbeck Canada Inc, Montreal, Quebec Canada; 3 Otsuka Canada Pharmaceutical Inc, Montreal, Quebec Canada; 4 JSS Medical Research, Montreal, Quebec Canada; 5 Department of Psychiatry, McGill University, Montreal, Quebec Canada; 6 ACCESS-Canada, 6625, boulevard LaSalle, Montreal, QC H4H 1R3 Canada

**Keywords:** Long acting injectable antipsychotics, Aripiprazole once-monthly, Global functioning, Adherence, Schizophrenia

## Abstract

**Background:**

With previously established efficacy of aripiprazole once-monthly injectable formulation (AOM) in pre-registration randomized controlled trials, the current study was designed to evaluate its effectiveness in patients treated for schizophrenia in regular clinical settings in Canada.

**Methods:**

Following their clinicians’ decision to prescribe AOM, 193 patients with a diagnosis of schizophrenia, were recruited from 17 Canadian community or hospital-based settings. The primary outcome of global functioning was assessed with the Global Assessment of Functioning Scale (GAF) at 3-month intervals for 1 year. Secondary outcomes (social and occupational functioning and illness severity) and adverse drug reactions (ADR) were also assessed.

**Results:**

A majority of the 169 evaluable patients were within the first 5 years of diagnosis (early phase). A linear mixed model analysis showed a significant main effect of time (Type III test *p* < 0.001) after adjusting for baseline GAF score, with a change in mean GAF scores from 49 at baseline to 61 at 12 months. No differences between early vs late phase were observed. Results on secondary outcome measures of function (Social and Occupational Functioning Scale) and illness severity (Clinical Global Impression-Severity Scale and Brief Psychiatric Rating Scale) were similar. Serious ADRs were observed in 29 (14.6%) patients and akathisia in 18 (9.1%) patients. At month-12, significant (≥7%) weight gain was observed in 25.7% (*n* = 27/105) of patients.

**Conclusions:**

Treatment with AOM is effective in improving symptoms and functioning in schizophrenia patients treated in regular clinical settings. Akathisia was infrequent while one quarter of patients gained clinically significant weight.

****Trial registration**:**

Unique identifier: NCT02131415. First posted: 06 May 2014.

**Electronic supplementary material:**

The online version of this article (10.1186/s12888-019-2103-x) contains supplementary material, which is available to authorized users.

## Background

Antipsychotic therapy is a cornerstone of treatment of patients with schizophrenia. However for a durable symptomatic remission, adequate antipsychotic medication is combined with evidence based psychosocial interventions [1]. Long periods of remission are almost necessary for a good functional outcome [2]. Relapse, which interrupts periods of remission, has serious consequences for the patient and the healthcare system [3, 4]. Relapse is predicted strongly by non-adherence to medication, substance abuse, poor pre-morbid adjustment and stress, especially in the early phase of the illness [5]. There is mounting recent evidence that long acting injectable (LAI) antipsychotics improve adherence primarily through more accurate monitoring at all stages of illness, including the “critical phase” of the first 2 to 5 years [6–10].

Despite the empirical evidence, which comes largely from population based and mirror-image studies, and clinical rationale supporting the benefits of LAIs in patients with schizophrenia, their use remains limited in routine clinical practice [11]. The use of LAIs is reported to have declined significantly after the introduction of second generation antipsychotic (SGA) medications in the 1990s [12]. This may have resulted from an assumption of better side effect profile of SGAs, especially extra-pyramidal side effects, being able to improve adherence.

The benefits of more consistent treatment and prevention of relapse is particularly crucial for the early phase of illness, when the potential for improved clinical outcomes is greater. It has been proposed that clinicians may be reluctant to recommend LAIs to patients with schizophrenia and related psychotic disorders as a clear choice of mode of receiving antipsychotic medications, especially early in the course of treatment [11, 13]. A recent survey of Canadian psychiatrists showed that, while there is considerable variation in the use of LAIs across Canada, the majority continue to use oral antipsychotics and that physicians may have had relatively limited experience with LAIs [13]. Barriers to using LAIs are different when examined from patient vs physician perspectives [11] and there is indeed inconsistency between reasons offered by patients [14] and those offered by physicians [13].

There are three second-generation LAI formulations currently available in Canada that are indicated for treatment of schizophrenia (risperidone, paliperidone, and aripiprazole). Aripiprazole is the first and only dopamine (D2) partial agonist to be developed as a LAI formulation, Aripiprazole once-monthly (AOM) [15–17]. AOM is indicated in Canada for the treatment of schizophrenia in adult patients, based on a pivotal randomized, double-blind, placebo-controlled trial of acutely relapsed adult patients as well as a mirror-image study that compared psychiatric hospitalisation rates before and after switching to AOM [18, 19]. In addition, the efficacy of AOM in the treatment of patients with schizophrenia was established, in part, on the basis of efficacy data from trials with the oral formulation of aripiprazole [20].

A recent randomized controlled trial (RCT), QUALIFY (*QUA*lity of *LI*fe with Abili*FY* Maintena), a multinational, open-label, 28-week, head-to-head study comparing AOM 400 mg to paliperidone palmitate (50–234 mg) found greater improvements in quality of life (Quality of Life Scale), disease severity (Clinical Global Impression — Severity [CGI-S]) and relative effectiveness (Investigator’s Assessment Questionnaire) in addition to better tolerability for AOM [21]. In a post-hoc analysis these results were observed to be particularly pronounced for younger patients of 35 years of age or less [21]. During an open-label 24-week extension of the QUALIFY study, effectiveness was maintained with small but continued improvements in quality of life and disease severity score vs paliperidone palmitate [22]. In a systematic review and indirect meta-analysis of relative efficacy and tolerability of AOM vs paliperidone palmitate in short-term RCTs, the results suggested relative advantages for AOM over paliperidone palmitate in the short-term treatment of schizophrenia. The authors adopted an indirect treatment comparison approach to overcome the lack of studies directly comparing the two treatments [23].

While the approval of a new medication is based on pivotal RCTs, results of such studies are not fully adequate in determining its true effectiveness in day to day use in clinical settings where the clinicians do not select patients based on strict inclusion criteria, established a-priori. Therefore, it is important to establish effectiveness and feasibility of using new medications such as AOM on a clinical population in routine practice that includes most, if not all, patients in need of treatment, including those with co-morbidities such as substance abuse. Furthermore, for most patients with a diagnosis of schizophrenia treated in regular clinical practice any treatment offered is often life-long, unlike RCTs.

Non-interventional post-approval clinical studies are the primary source of valid information about real-life effectiveness and safety of targeted patient populations and provide data that is more generalized to real-life. *Re*al-*Li*fe Assessment of *A*bilify *M*aintena (ReLiAM) in schizophrenia is a naturalistic, non-interventional prospective cohort study to assess the effectiveness of AOM for patients treated for schizophrenia in regular clinical settings in Canada.

The primary objective was to assess the impact of treatment with AOM over a period of 12 months on the global functional status of patients with schizophrenia, as measured by the Global Assessment of Functioning. While there were several secondary objectives, here we report results on only a select number of these that are clinically relevant.

## Methods

### Design

This was a non-interventional, Canadian, prospective cohort, multi-site study in patients treated with AOM for schizophrenia for up to 24 months. Study assessments took place during the patient’s regular assessments or injection visits that were part of routine care. However, a schedule of 9 visits (baseline and months 3, 6, 9, 12, 15, 18, 21 and 24) was recommended. The study was originally designed to assess outcome at the end of 2 years. However, it was terminated before all patients completed the 2 years, following the positive results from the pre-planned interim analyses, which was to be conducted after at least 50% of the initially planned number of patients had completed the 12-month assessment. This decision was made by the study sponsor in consultation with the investigators. The one-year results for the entire population are reported herein.

### Population

Patients were recruited from 17 Canadian community or hospital-based clinical settings, selected as a representative sample of clinical environment for practice of Canadian psychiatrists who treat patients with schizophrenia spectrum disorders. One hundred and ninety-three patients were enrolled based on the following criteria:

• Inclusion criteria: Patients diagnosed with schizophrenia; at least mildly ill (CGI-S score of ≥3), age 18 years (19 for patients from British Columbia) or older, fluent in English and/or French, able to provide informed consent and the treating psychiatrist had decided, prior to and independently of enrolment in the study, to prescribe AOM for treatment.

• Exclusion Criteria: The patient did not comprehend the informed consent, had contraindications to the use of AOM as specified in the Canadian Product Monograph, had previously received one or more doses of AOM, presented a significant suicidal risk as judged by the investigator, or was a pregnant or lactating female.

All patients signed an informed consent before any study related procedures were performed. Each site obtained ethics approval from their local institutional ethics board.

### Assessments and measures

Demographics such as age, gender, race, and clinical characteristics such as, body weight (kg), and treatment doses of AOM were recorded. Percentage medication adherence was calculated as the number of total injections divided by times of exposure to treatment in months × 100.

The primary outcome measure was the score on Global Assessment of Functioning Scale (GAF) which evaluates psychological, social and occupational functioning in addition to symptoms [24]. The Social and Occupational Functioning Scale (SOFAS) [25] is derived from GAF and evaluates the patient’s level of social and occupational functioning independent of the severity of the patient’s psychological symptoms and was used as a secondary outcome measure in this study. Scores for both GAF and SOFAS range from 0 to 100, where a high score represents superior functioning and a low score represents poor functioning. The Clinical Global Impression-Severity Scale (CGI-S) [26] provides an aggregate score for clinical severity based on the clinician’s knowledge of the patient and scores range from 1 (Normal, not at all ill) to 7 (Among the most extremely ill patients). The Brief Psychiatric Rating Scale (BPRS) [27], (18 items, each scoring from 1 (not present) to 7 (extremely severe), with a possible total score in the range of 18–126), was used to assess more detailed psychopathology and its severity.

Remission was defined as a score of 3 or less on the following BPRS items for at least 6 months i.e. 3 consecutive visits (that was extended to 4 consecutive visits from the original 3 if one of the scheduled visit was missing): grandiosity, suspiciousness, unusual thought content, hallucinatory behavior, conceptual disorder, mannerisms, and blunted affect [28].

Relapse was assessed for patients who had achieved remission at some point (including baseline). Patients (following remission) were defined to be in relapse if they experienced (a) worsening of psychiatric symptoms that led to their hospitalization or withdrawal from study, or; (b) an increase equal to or greater than 1 point in the CGI-S compared to the last available measurement that resulted in a score ≥ 4.

Both serious adverse drug reactions (ADR) such as suicidality as well as non-serious ADR such as mild nausea were recorded.

### Antipsychotic pharmacotherapy

The decision to treat the patient with AOM had to be reached independently of, and prior to recruitment in the study and treatment had to be in accordance with the approved Canadian Product Monograph and as per the decision of the treating physician. All patients were treated with AOM administered once monthly by the treating physician with the recommended dose of 400 mg. As per product monograph, dose reduction to 300 mg was recommended in special populations or in case of ADRs. In addition, all patients received the respective model of additional care available within each clinic where they were treated.

### Statistical analysis

Given that this is a single cohort study the sample size requirements were based on the precision of the estimate for the primary study endpoint, namely the change in GAF from baseline to 12 months, as assessed by the width of the 95% confidence interval (CI). A 95% CI width of ±10 to 20% of the point estimate was considered to provide adequate precision. In the study by Christensen et al. (2006) [29] the mean total GAF score increased from 39.4 to 46.2 after 180 days of treatment with oral aripiprazole. Conservatively assuming that, at 12 months of treatment, the mean change in GAF from baseline would be approximately 7.0 and a coefficient of variation of 1, the required 95% CI would be between ±0.7 and 1.4. Assuming a width of 1.0, the minimum sample size requirement was set at 189 patients.

The primary outcome analysis population was comprised of all patients who were enrolled in the study, had received at least one dose of AOM, and had at least one follow-up assessment visit. All patients that received at least one dose of AOM were included in the safety assessments. Descriptive statistics including the mean and SD for continuous scale variables and frequency distributions for categorical scale variables, were produced for all study variables. Kaplan-Meier survival analysis was used to assess probability of remission and relapse. Linear mixed models adjusting for baseline scores were used for the primary analysis in this study (SAS, version 9.4; SAS Inc., Cary, NC, USA). For exploratory purposes, a stratified post-hoc analysis was conducted based on time since first diagnosis, namely patients with ‘early-phase’ psychosis (≤ 5 years) and ‘later-phase’ psychosis (> 5 years). Between-group (‘early-phase’ vs. ‘later-phase’ psychosis) differences in outcomes were also calculated using linear mixed models. All statistical tests were 2-sided and a *p*-value of 0.05 or less was considered significant.

## Results

**Fig. 1:**
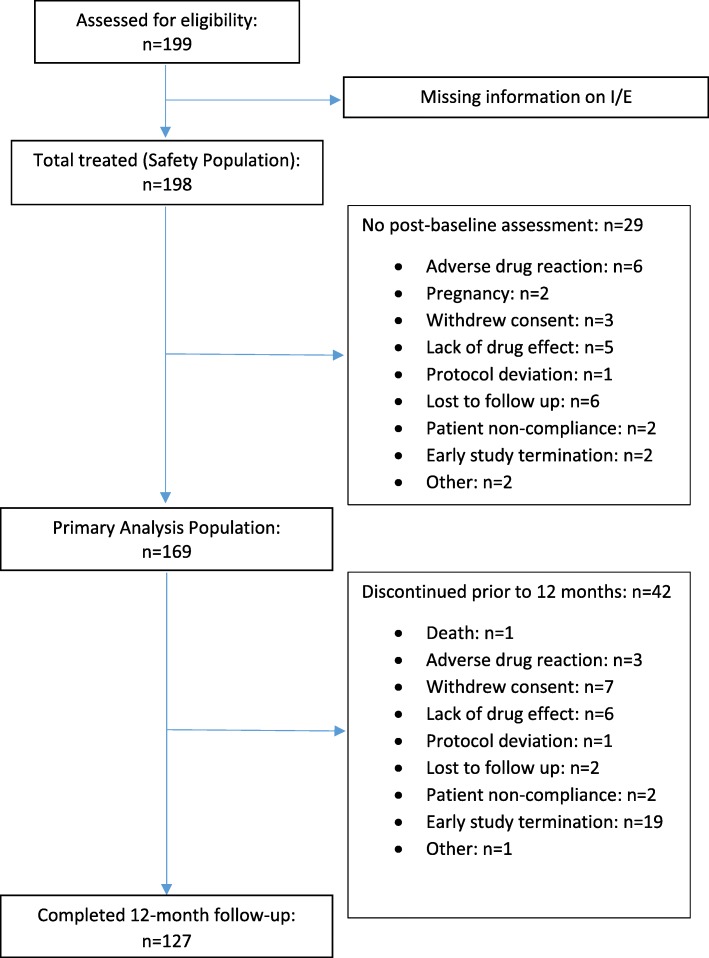
Consort flow diagram

At 17 sites in Canada, 199 patients were assessed for eligibility. Of those, 169 patients met all eligibility criteria, took at least one dose of AOM, and had a baseline and at least one post-baseline assessment; this constituted the primary analysis population of this study. Forty-two patients discontinued the study prior to 12 months; details are presented in Fig. 1. Patients’ demographic and clinical characteristics are shown in Table 1.

**Table 1:** Baseline characteristics of the sample

	Total sample^a^ (*n* = 169)	Early-phase (*n* = 108)	Later-phase (*n* = 59)
Male gender, n (%)	114 (67.5%)	75 (69.4%)	38 (64.4%)
Age at consent (years), mean (SD)	32.9 (12.2)	28.0 (9.4)	41.6 (11.9)
AOM mean dose (mg), mean (SD)	371.6 (50.2)	371.3 (53.0)	371.2 (45.7)
GAF score, mean (SD)	48.7 (12.6)	49.1 (13.6)	48.5 (10.6)
SOFAS score, mean (SD)	50.1 (12.3)	50.5 (13.0)	49.9 (11.0)
CGI-S score, mean (SD)	4.0 (0.8)	4.0 (0.9)	4.1 (0.6)
BPRS score, mean (SD)	39.8 (11.3)	39.8 (12.7)	39.6 (8.2)

^a^At enrollment, 2 patients could not be characterized as early or later phase

One hundred and eight patients (63.9%) were classified as ‘early-phase psychosis’ and 59 (34.9%) as ‘later-phase psychosis’. At enrolment, 2 patients could not be characterized as early or later phase due to lack of reliable information. Adherence with the prescribed treatment while patients were on treatment was high with a mean of 1.04 injections (95% CI: 1.02–1.06) administered per month, Additional file 1: Table S1.

### Primary outcome; GAF

**Fig. 2:**
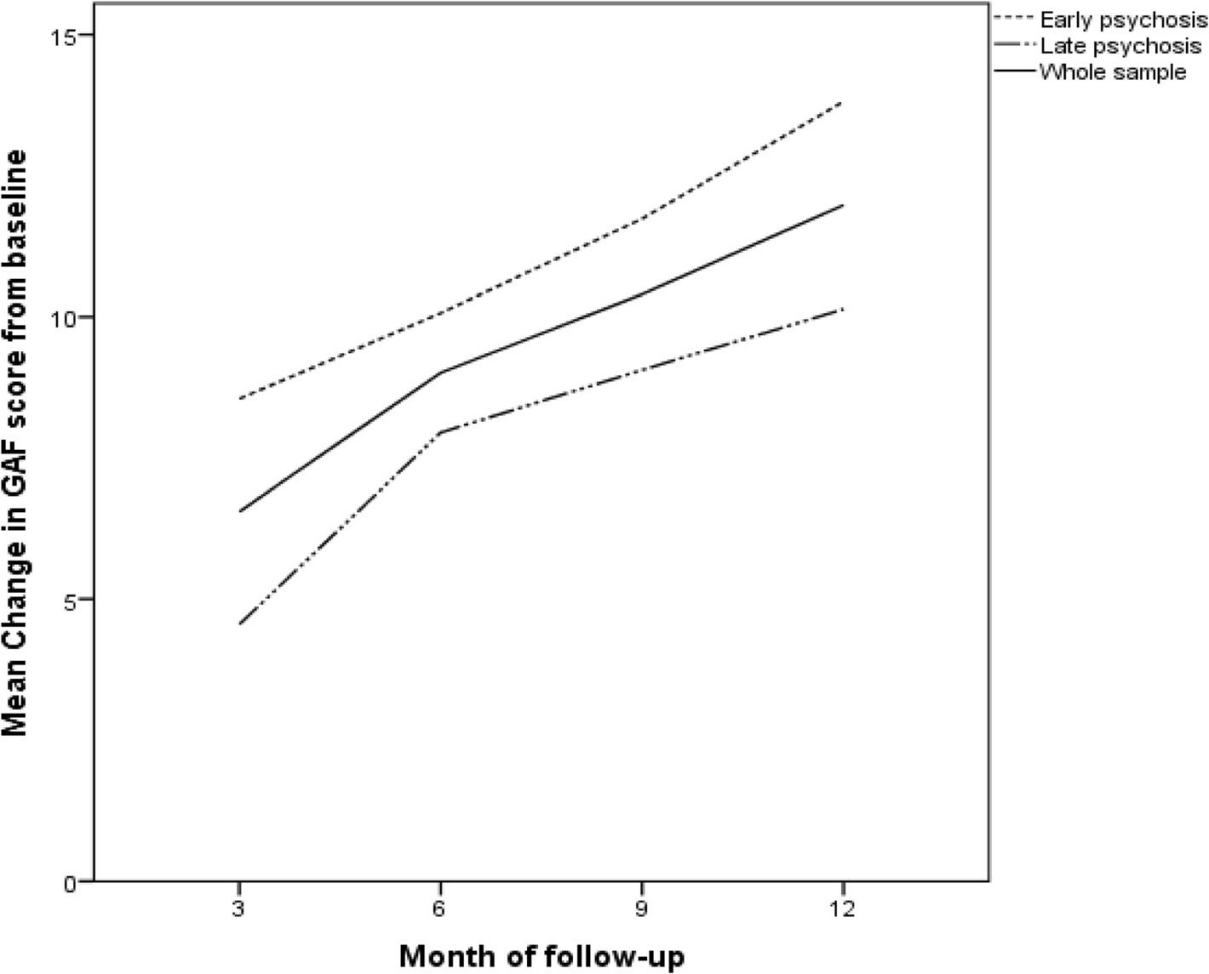
Mean change in Global Assessment of Functioning (GAF) from baseline to month 12 (mean baseline GAF score for whole sample = 48.7, early psychosis = 49.1, late psychosis = 48.5)

The linear mixed model analysis showed a significant main effect of time (*p* value < 0.001) after adjusting for baseline GAF score. Mean GAF scores changed from 49 at baseline to 61 at 12 months. This represents a 2-category improvement from “*serious symptoms OR any serious impairment in social, occupational or school functionin*g” at baseline to “*some mild symptoms OR some difficulty in social, occupational or school functioning, but generally functioning pretty well, has some meaningful interpersonal relationships*” [24] at month 12. In the post-hoc analysis a trend was observed in the between subject effects for early vs late psychosis favoring early psychosis (*p* = 0.063), Fig. 2.

### Secondary outcomes

#### Other functional measure; SOFAS

Consistent with the GAF findings, a significant main effect of time (Type III test *p* value < 0.001) was found after adjusting for baseline SOFAS scores. Patients’ mean SOFAS scores increased from 50 at baseline, which corresponds to the category “*serious impairment in social, occupational or school functioning*” to a mean score of 61 at 12 months, implying “*some difficulty in social, occupational or school functioning, but generally functioning pretty well, has some meaningful interpersonal relationships*”. This represents a 2-category improvement. In the post-hoc analysis, there was no significant difference in the between subject effect of early vs late psychosis (*p* = 0.073).

### Illness severity

#### CGI-s

At baseline, patients were “moderately ill” on the CGI-S; a mean score of 4. At month 12 the score dropped by one category to 3, corresponding to mildly ill. Mixed-model ANOVA, after adjusting for baseline CGI-S score, showed this main effect of time to be significant (Type III test *p* value < 0.001). The improvement was significantly greater in early vs late psychosis (*p* = 0.005).

#### BPRS

At baseline, patients were “moderately ill” based on BPRS scores (M = 39.8, SD = 11.3). At month 12 the score dropped significantly to 29.4 (Type III test p value < 0.001), showing a significant main effect of time after adjusting for baseline score. There was no significant difference in the between subject effect of early vs late psychosis (*p* = 0.084).

#### Remission and relapse

Remission was calculated in two different ways, proportion of patients in remission at any time during the 12-month period including those who were in remission at the time of entry into the study and those who achieved remission only during the treatment with AOM while in the study. Based on Kaplan-Meier survival analysis the probability for being in remission at 12 months for the former was 54.3% (95%CI: 46.3–62.7%) (including patients who were in remission at baseline) and 36.7% (95%CI: 29.3–45.4%) for the latter, those who achieved remission while in the study.

The probability of relapse was calculated for those who were in remission and, therefore, the time to be at risk of relapse varied depending on when the patients went into remission. We are, therefore, reporting probability of relapse at 3, 6 and 9 months. These rates were 5.4% (95% CI: 2.3–12.6%) at 3 months, 8.9% (95% CI: 4.5–16.9%) at 6 months and 21.1% (95% CI: 13.6–31.8% at 9 months post-achieving remission.

### Adverse drug reactions

For assessment of safety indices, the sample size was 198. During the 12-month study period, a total of 137 patients (69.2%) experienced a total of 366 adverse events. Of these, 45 were deemed “not related” to AOM, 53 “were possibly related”, and 134 were “probably related”. Following those events, AOM was either discontinued (*n* = 32 events), dose changed (*n* = 46), new concomitant medication and/or non-drug therapy given (*n* = 49), concurrent concomitant medication switched (*n* = 13), hospitalization prolonged (*n* = 41) or no action was taken (*n* = 231). Serious ADRs were observed in 29 (14.6%) patients for a total of 52 events. Akathisia occurred in 18 (9.1%) patients. The average weight was 85.7 (SD = 22.1) kg at baseline and 89.2 (SD = 21.7) at one-year which was not significantly different; *t* (206) = − 1.189, *p* = 0.118. At month-12, significant (≥7%) weight gain was observed in 25.7% (*n* = 27/105) of patients with no difference between groups; early psychosis: 27.1% (*n* = 19/70) and late psychosis: 22.9% (*n* = 8/35). Due to the naturalistic nature of the study, in the absence of protocol requirement, weight was not systematically measured for all patients at all time points. Therefore, weight data are missing for some patients.

## Discussion

ReLiAM is the first naturalistic, non-interventional Canadian long-term study to be reported for patients with schizophrenia treated with AOM under real-life and regular clinical service conditions.

The study demonstrated that treatment with AOM resulted in significant improvements, over a one-year period, in both functioning and symptoms in patients with schizophrenia. GAF was selected as the primary endpoint to capture an overall assessment of patient functioning in a single score that reflects psychiatric symptoms, and to a lesser extent social functioning. For assessing global functional outcome without the impact of symptoms, we had chosen the SOFAS as a secondary outcome. Both outcome measures of functioning showed a progress from serious to mild impairment. Severity of illness was significantly reduced from moderate to mild as detected by both CGI-S and BPRS and also indicated by relatively high rates of remission of both positive and negative symptoms. These favourable clinical outcomes during the 12-month study were observed despite the fact that at the time of recruitment, patients were relatively stable, although only a small proportion were in full remission. This is in line with recently published studies showing AOM to be efficacious across the spectrum of schizophrenia symptoms [30] and also observed in studies conducted with other atypical antipsychotics [31]. This could also be related to the fact that LAI medication assures adherence as long as the patient is receiving the injection.

Patients recruited in the present study had received a diagnosis of schizophrenia. While ReLiAM was not designed to evaluate differential outcomes in patients in the early (five or less years since diagnosis) vs late phase illness (more than 5 years since diagnosis), we conducted a post-hoc examination of this question. Our results suggest that there were no significant differences observed in the impact on functional outcome between the two groups, although there was a trend towards greater benefit for early phase patients. Only the overall assessment of clinical severity, as assessed by CGI, showed a significant difference between the two groups with greater improvement seen in the early phase patients. The lack of difference between early vs late phase patients in functional outcome may appear inconsistent with previous studies of AOM showing numerically greater symptom and functioning improvements in younger patients [21]. However, the latter report was based on a study with a very different design (RCT comparing AOM with paliperidone palmitate LAI), using different measures of outcome (Quality of Life Scale in the QUALIFY study vs GAF and SOFAS in the current study) and most importantly, a different definition of “early” and “late” phase psychosis. In the QUALIFY study the post-hoc analysis based on age used a cut-off of 35 while in the present study we used a cut-off of 5 years since diagnosis. The former provides, at best, an indication that younger patients may have responded better and it is implied that they would be in an earlier phase of the illness. However, many patients who were 35 years old or younger would have been much past 10 years since diagnosis given that the mean age of onset of psychosis is around 22 and 25 years for men and women, respectively [32, 33]. In our study, the classification is more closely aligned to definition of early phase, often referred to as ‘critical phase’ of the first 5 years [34] and does not rely on age as the means for classification.

In the present study, the better outcome reported on CGI in early-phase patients is not matched by the other measure of symptom severity, i.e., BPRS total scores. This may imply some bias in the clinicians’ rating of overall severity for their own patients in the early phase, who they expect to show better response, especially in the absence of a control.

While mean overall increase in body weight by the end of the one-year of this study was not statistically significant, one quarter of patients did show clinically significant (≥7%) weight gain. However, this proportion of patients with significant weight gain was not different between the stages of psychosis; early vs late. This proportion is lower than that reported in a recent study [35] where oral aripiprazole was associated with a higher proportion of first-episode psychosis patients showing clinically significant weight gain. This difference could be explained by differences in patient characteristics, with the latter study sample comprised of patients with first episode psychosis with little or no previous exposure to antipsychotic medications. Such young patients are known to be particularly vulnerable to weight gain with the use of most psychotropic medications [36]. Further, the current study used LAI aripiprazole while the Malla et al. (2016) study used oral aripiprazole. It is not clear whether there are any significant differences between oral and long acting preparations of aripiprazole in relation to weight gain.

### Limitations

Although this study was designed as a demonstration of effectiveness of AOM in regular clinical settings, the limitations of this design cannot be overlooked. These biases include issues related to sampling of sites as well as patients within each site, lack of consideration of variation in other treatments, variation in external environment etc. Clinics were included not based on a random sampling but more through invitation to clinics known to be involved in treatment of psychosis, including early intervention services. Within each clinical setting patients were first prescribed AOM by the treating psychiatrist before being enrolled in the study. Clinician’s initial choice may have included some implicit or explicit bias in selecting certain types of patients who the clinician believed to likely benefit from a LAI. This is, however, likely to have selected patients who were non-adherent and not necessarily those with an inherently better outcome. Most patients receive some other interventions as part of their treatment although there is a large variation in the quantity and quality of the interventions provided. These other interventions are mostly psychosocial treatments such as, cognitive behavioural therapy (CBT), family intervention, case management etc. All of these interventions are known to positively influence outcome by either reducing residual symptoms (in the case of CBT), reducing risk of relapse (for family intervention), greater retention in and higher adherence to treatment and improving general outcomes (early intervention in psychosis) [37]. The treatment environments also show a large variation with early psychosis patients likely to be treated in better-resourced early intervention services. All of these are likely to influence outcome. Although the study was designed not to include a control condition, the outcome could be seen as a natural course of the illness. This, however, is very unlikely as the natural course of the illness is predominantly one of recurrences and low rates of remission if not treated. It is reasonable to assume that the outcomes are likely to have been influenced by consistent availability of anti-psychotic medication through the LAI, assuring adherence and, thereby, facilitating remission and reducing the risk of relapse. In fact, the risk of relapse of around 21% is similar to that reported for a highly intensive early intervention service with high rates of adherence to medication [38].

## Conclusion

Our results show that AOM is an effective treatment in all phases of schizophrenia, is fairly well tolerated and is associated with improvement in both clinical indices as well as global functional outcome. The efficacy of the drug having already been established, this study demonstrated effectiveness of this LAI for schizophrenia patients treated in regular community and hospital out-patient clinical settings. While LAIs do not treat non-adherence, their use does ensure better monitoring as demonstrated by high adherence to receiving the LAI. Regarding tolerability, a small proportion of patients (9%) experienced akathisia whereas 26% gained clinically significant weight. These results are encouraging and should inform and influence practice patterns of clinicians treating patients with schizophrenia in all phases. Such naturalistic study overcomes the limitations of RCTs, which often recruit patients limited to strict inclusion and exclusion criteria, who do not represent the typical heterogeneous patient population treated in the real-life setting.

## Additional file

Additional file 1: **Table S1.** Adherence rate to AOM from first to last injection. (DOCX 14 kb)

## Abbreviations

ADR: adverse drug reactions
AOM: Aripiprazole once-monthly
BPRS: The Brief Psychiatric Rating Scale
CGI-S: Clinical Global Impression — Severity
GAF: Global Assessment of Functioning Scale
LAI: Long acting injectable
QUALIFY: *QUA*lity of *LI*fe with Abili*FY* Maintena
RCT: Randomized controlled trial
ReLiAM: *Re*al-*Li*fe Assessment of *A*bilify *M*aintena
SGA: Second generation antipsychotic
SOFAS: The Social and Occupational Functioning Scale
