# Supplementary material for: Real-life assessment of aripiprazole monthly (Abilify Maintena) in schizophrenia: a Canadian naturalistic non-interventional prospective cohort study
Source: BMC Psychiatry. 2019 Apr 16;19:114. doi: 10.1186/s12888-019-2103-x (PMC6469112; doi:10.1186/s12888-019-2103-x)
Supplement: Supplementary file 1 — Table S1. Adherence rate to AOM from first to last injection. (DOCX 14 kb) [file 12888_2019_2103_MOESM1_ESM.docx]

Additional file 1: Table S1. Adherence rate to AOM from first to last injection

| Adherence Rate (%) | First to Last Injection^1^ | | |
| --- | --- | --- | --- |
|  | n |  | 169 |
|  | Mean |  | 103.8 |
|  | 95% Confidence Interval for Mean | Lower Bound  Upper Bound | 101.8  105.9 |
|  | Median |  | 105.9 |
|  | Standard Deviation |  | 13.42 |
|  | Minimum |  | 37.4 |
|  | Maximum |  | 164.4 |

^1^Adherence rate = Number of total injections received/Length of treatment exposure (months)*100.
